# Characterization of three plant biomass-degrading microbial consortia by metagenomics- and metasecretomics-based approaches

**DOI:** 10.1007/s00253-016-7713-3

**Published:** 2016-07-14

**Authors:** Diego Javier Jiménez, Maria Julia de Lima Brossi, Julia Schückel, Stjepan Krešimir Kračun, William George Tycho Willats, Jan Dirk van Elsas

**Affiliations:** 1 Department of Microbial Ecology, Groningen Institute for Evolutionary Life Sciences, University of Groningen, Nijenborgh 7, 9747 AG Groningen, The Netherlands; 2 Department of Plant and Environmental Sciences, University of Copenhagen, Thorvaldsensvej 40, Frederiksberg C 1871, Copenhagen, Denmark

**Keywords:** Enzyme cocktails, Metagenomics, Metasecretome, Microbial consortia, Plant biomass

## Abstract

The selection of microbes by enrichment on plant biomass has been proposed as an efficient way to develop new strategies for lignocellulose saccharification. Here, we report an in-depth analysis of soil-derived microbial consortia that were trained to degrade once-used wheat straw (WS1-M), switchgrass (SG-M) and corn stover (CS-M) under aerobic and mesophilic conditions. Molecular fingerprintings, bacterial 16S ribosomal RNA (rRNA) gene amplicon sequencing and metagenomic analyses showed that the three microbial consortia were taxonomically distinct. Based on the taxonomic affiliation of protein-encoding sequences, members of the *Bacteroidetes* (e.g. *Chryseobacterium*, *Weeksella*, *Flavobacterium* and *Sphingobacterium*) were preferentially selected on WS1-M, whereas SG-M and CS-M favoured members of the *Proteobacteria* (e.g. *Caulobacter*, *Brevundimonas*, *Stenotrophomonas* and *Xanthomonas*). The highest degradation rates of lignin (~59 %) were observed with SG-M, whereas CS-M showed a high consumption of cellulose and hemicellulose. Analyses of the carbohydrate-active enzymes in the three microbial consortia showed the dominance of glycosyl hydrolases (e.g. of families GH3, GH43, GH13, GH10, GH29, GH28, GH16, GH4 and GH92). In addition, proteins of families AA6, AA10 and AA2 were detected. Analysis of secreted protein fractions (metasecretome) for each selected microbial consortium mainly showed the presence of enzymes able to degrade arabinan, arabinoxylan, xylan, β-glucan, galactomannan and rhamnogalacturonan. Notably, these metasecretomes contain enzymes that enable us to produce oligosaccharides directly from wheat straw, sugarcane bagasse and willow. Thus, the underlying microbial consortia constitute valuable resources for the production of enzyme cocktails for the efficient saccharification of plant biomass.

**Electronic supplementary material:**

The online version of this article (doi:10.1007/s00253-016-7713-3) contains supplementary material, which is available to authorized users.

## Introduction

Plant biomass is an important source of energy that is stored in the form of complex polysaccharides, primarily hemicelluloses and cellulose. The transformation of these polymers into sugars enables downstream applications such as the production of biofuels. The saccharification process is currently carried out by (thermochemical) pretreatment followed by the use of a mixture of microbial enzymes (e.g. lytic polysaccharide monooxygenases, xylanases, arabinofuranosidases, cellobiohydrolases, endoglucanases and β-glucosidases) that can work synergistically (Meyer et al. 2009; Hasunuma et al. 2013). Plant waste sources that are used for the production of second generation of biofuels include agricultural by-products (e.g. sugarcane bagasse), wood residues and non-food energy crops, such as switchgrass. Such are attractive as they do not seem to compete with food production (Sims et al. 2010; Limayem and Ricke 2012).

The leading industrial source of cellulase cocktails is *Trichoderma reesei*. Several strains exist and their secretomes have been widely used to develop commercial cocktails for plant biomass hydrolysis (e.g. Celluclast 1.5 L, Cellic CTec2 and HTec2 from Novozymes). However, *T. reesei* secretomes are dominated by cellobiohydrolases (CBHs) and endoglucanases, with only low quantities of xylanases, lytic polysaccharide monooxygenases (LPMOs), and β-glucosidases being produced. Hence, addition of such enzymes is thought to improve the hydrolytic efficiency (Mohanram et al. 2013). For instance, Gao et al. (2011) showed that the addition of defined hemicellulases (e.g. β-xylosidases, α-arabinofuranosidases and α-glucuronidases) from *Clostridium thermocellum*, *Geobacillus thermodenitrificans*, *Geobacillus stearothermophilus* and *Dictyoglomus turgidum*, to a core cellulase cocktail from *T. reesei* and *Aspergillus niger*, enhances the saccharification of pretreated corn stover. Typically, in biorefinery processes, Celluclast 1.5 L (1,4-(1,3:1,4)-β-D-glucan 4-glucano-hydrolase) is supplemented with a β-glucosidase from *A. niger* (Merino and Cherry 2007). Moreover, Cellic CTec2 includes cellulases, high levels of improved β-glucosidases with less glucose inhibition, hemicellulases and LPMOs. In industry, it is recommended to dose the Cellic CTec2 in accordance with the level of cellulose in the substrate. If (pretreated) plant biomass contains an appreciable amount of hemicellulose, it is advised to combine Cellic CTec2 with HTec2 (endoxylanases) to boost cellulose hydrolysis (Cannella and Jørgensen 2014; Rodrigues et al. 2015).

Given the complexity of the required enzymes, efficient plant biomass hydrolysis by microbial consortia, instead of single strains, has been proposed (Cheng and Zhu 2012). One disadvantage of this strategy is that the monosaccharides released from plant biomass are often rapidly assimilated by co-occurring microorganisms. To overcome this hurdle, extracellular enzymes may be harvested from the microbial consortia and applied directly onto the plant biomass (Gladden et al. 2011a; Park et al. 2012). Enrichments of lignocellulolytic microbes from soils have been performed with switchgrass (SG), wheat straw (WS) and corn stover (CS) as the sole sources of carbon (DeAngelis et al. 2013; Jiménez et al. 2014a; Brossi et al. 2015). Such plant biomass is known to not only contain recalcitrant polysaccharides, but also (easily degradable) small soluble substrates (e.g. oligosaccharides). These increase the proliferation of opportunistic microorganisms that cannot deconstruct the lignocellulosic structures. To remove such soluble substrates, washes of the plant biomass with water and ethanol have been proposed (Gladden et al. 2011a). Moreover, biological pretreatment can be based on living organisms or on enzyme cocktails. The former is exemplified by the use of white-rot basidiomycetes such as *Phanerochaete chrysosporium* and *Trametes versicolor* (Pinto et al. 2012; Wan and Li 2012). The latter makes use of commercial enzyme cocktails (as explained earlier). However, biological pretreatments using (enzymes from) microbial consortia offer alternatives that have so far been poorly explored.

Metagenomics- and metatranscriptomics-based approaches have been increasingly used to study lignocellulolytic microbial consortia (Wongwilaiwalin et al. 2013; Simmons et al. 2014). Comparison of metagenomic sequences with data stored in the “Carbohydrate-Active Enzyme database” (CAZy) (Lombard et al. 2014) allows for evaluation of the metabolic potential in the deconstruction of plant polysaccharides. Recently, Jiménez et al. (2015a) unveiled such potential in two microbial consortia selected on wheat straw. Significant enrichments of genes encoding GH2, GH43, GH92 and GH95 family proteins were found. In taxonomic terms, the genes were mostly affiliated with those present on the genomes of *Sphingobacterium*, *Bacteroides*, *Flavobacterium* and *Pedobacter* species.

Here, we used an enrichment process in two stages, i.e. (1) enriching biodegrader soil-derived microbial consortia on wheat straw, switchgrass and corn stover (Brossi et al. 2015) and then (2) re-using the partially degraded substrate as the carbon source for a second growth step with the same microbial consortia. We hypothesised that the once-used plant biomass specifically selected for microbes with high capacities to degrade the more complex plant polysaccharides as well as lignin. We thus presumed the biological pretreatment removed the easily degradable substrates from the three plant biomass materials and studied how the microbial consortia changed along the two steps in the enrichment process. The main aim of this study was to characterize these selected “second-phase” microbial consortia by lignocellulose consumption profiles, metagenomics (taxonomic and CAZy profiling) and extracellular enzymatic activities using a new generation of versatile chromogenic substrates (Kračun et al. 2015).

## Methods

### Microbial consortia cultivated on once-used plant biomass

**Fig. 1:**
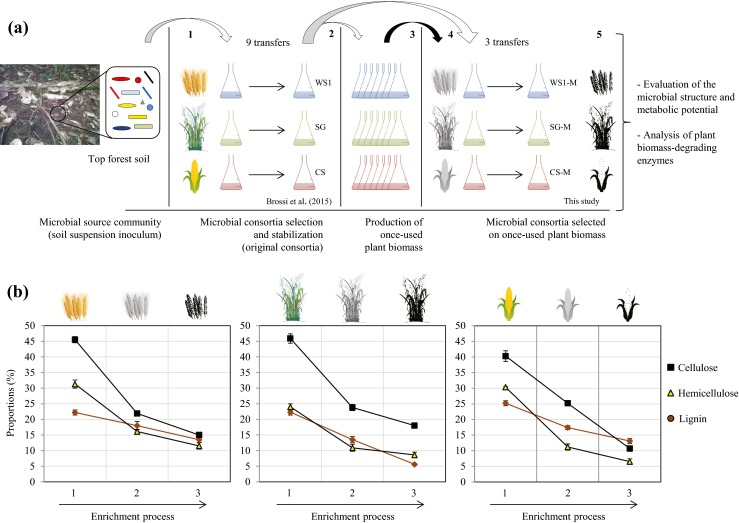
**a** Schematic representation of the enrichment strategy. *1* Inoculation of microbial cells from forest soil samples; *2* Inoculation of soil-derived microbial consortia in 10 flasks per treatment in order to produce the once-used plant biomass; *3* Harvesting of plant biomass for the subsequently enrichment process; *4* Inoculation of microbial cells from the original microbial consortia in the flasks that contained once-used plant biomass and *5* Harvesting of plant biomass remains, microbial cells for metagenome analysis, and metasecretomes to evaluate enzymatic activities. **b** Proportions of lignin, cellulose and hemicellulose of the original (raw), once-used (after growth of the original microbial consortia) and remaining plant biomass (after growth of the “second-phase” microbial consortia). *1* original; *2* once used and *3* remaining plant biomass (twice used)

Three enrichment cultures were established with soil as a microbial source and three plant biomass samples (wheat straw, switchgrass and corn stover) as unique carbon and energy sources (Fig. 1a). The plant waste materials were air-dried before cutting into pieces of about 1-mm length and added to the enrichment medium described as follows. Ten randomly taken soil samples of 10 g each were collected from a forest (0 to 10 cm depth) in Groningen, The Netherlands (53.41 N; 6.90 E) in September 2013. Selection of the stable soil-derived microbial consortia has been reported before (Brossi et al. 2015). Briefly, cell suspensions were prepared by adding 10 g of mixed soil to 250-ml flasks containing 10 g of sterile gravel in 90 ml of 0.9 % saline solution (NaCl). The flasks were shaken for 20 min at 250 rpm. Aliquots (150 μl) of soil suspension were added to triplicate flasks containing 15 ml of mineral salt medium (MSM, pH 7.2), with 1 % of plant biomass and trace mineral and vitamin (TMV) solutions (Jiménez et al. 2014a). Flasks were incubated at 28 °C in oxic conditions (with shaking at 150 rpm). Once systems reached high bacterial cell density (7–8 log cells/ml, between 5 and 6 days), aliquots (15 μl) of microbial suspension were transferred to fresh medium (diluted 1000 times). These procedures were repeated nine times.

Once soil-derived microbial consortia had been bred (transfer 9: WS, SG and CS), production of once-used lignocellulose was performed as described: cells from each consortium (25 μl of microbial suspension) were introduced into 25 ml of fresh plant biomass (1 %) containing medium (a single batch: 10 flasks per consortium), and, subsequently, the flasks were incubated at 28 °C, 150 rpm (pH 7.2). After microbial growth was achieved (6 days), the enriched cultures were filtered through Whatman paper (grade 1) and the plant biomass remains were washed three times with sterile water and dried at 65 °C for 3 days. The dry plant biomass was recovered, avoiding scratching the filter, and then immediately used as a carbon source in the following enrichment stages. Finally, cells from the previous soil-derived microbial consortia (transfer 9) were reintroduced into triplicate flasks of 25 ml of MSM + TMV containing 1 % of the once-used plant biomass. Selection of stable microbial communities, on the once-used substrates, was performed by three sequential transfers (denoted as transfers 11, 12 and 13) using the dilution-to-stimulation approach as indicated earlier (Fig. 1a). A negative control without microbial source was also set up. Samples were taken from each microbial consortium at transfer 13 and stored with 20 % of glycerol at −80 °C.

### Substrate weight loss and composition of each plant biomass

At the end of transfers 11, 12 and 13 in the newly selected microbial consortia (wheat straw: WS1-M; switchgrass: SG-M and corn stover: CS-M), the weight of the residual plant biomass was measured and compared to a control treatment without the inoculum. The plant biomass was washed thoroughly with sterile water (three times) in order to remove the microbial biomass and proteins. The percentage of weight loss was defined as the ratio of the weight loss compared to the initial weight (%) as calculated by the following formula: substrate weight loss (%) = [(*a* − *b*)/*c*] × 100; where *a* is the residual control substrate weight, *b* is the residual substrate weight and *c* is the total substrate weight. To determine the composition of each substrate (plant biomass) before and after growth of each microbial consortium, we used Fourier transformed infrared (FTIR) spectroscopy (Adapa et al. 2001). Quantification of the proportions of cellulose, hemicellulose (i.e. xylan from birchwood as the proxy) and lignin was performed according to Brossi et al. (2015). Degradation rates were expressed as the ratio of the proportions of each component in the substrate after incubation compared to the proportions of each component before incubation, as follows: degradation rate (%) = [(*a* − *b*)/*a*] × 100; where *a* is the proportion of component in the substrate before incubation and *b* is the proportion of component in the substrate after incubation. Statistical comparisons between degradation rates were performed using one-way ANOVA (Tukey’s test).

### Total microbial DNA extraction and PCR-DGGE

DNA was extracted from each microbial consortium (in triplicate) using the UltraClean Microbial DNA Isolation Kit (MoBio Laboratories Inc., Carlsbad, CA, USA) according to the manufacturer’s instructions. Bacterial community structures, in the soil-derived microbial consortia (original- transfer 9) and in the final microbial consortia cultivated on the once-used plant biomass (selected- transfer 13: WS1-M, SG-M and CS-M), were evaluated by PCR-denaturing gradient gel electrophoresis (PCR-DGGE). Primer sequences, PCR and DGGE conditions were previously reported (Jiménez et al. 2014a; Brossi et al. 2015). Fingerprinting results were analysed using GelCompar software (Applied Maths, Sint-Martens-Latem, Belgium). Thus, presence/absence band patterns were converted in Jaccard dissimilarity matrices for non-metric multi-dimensional scaling (nMDS) using Primer6 (PrimerE, Ivybridge, UK).

### Bacterial 16S rRNA gene amplicon sequencing and data processing

Bacterial community structures, in the microbial source (forest soil), the original microbial consortia (WS1, SG and CS) and in the final microbial consortia cultivated on the once-used plant biomass (WS1-M, SG-M and CS-M), were evaluated by Illumina MiSeq (2 × 300 bp) amplicon sequencing. Briefly, PCR reactions were performed using the primer set FP16S (5′-TGYCAGCMGCCGCGGTA-3′) and RP16S (5′-CCGYCAATTYMTTTRAGTTT-3′) that targets regions V4–V6 of the 16S ribosomal RNA (rRNA) bacterial gene. Twenty-five-microlitres PCR reactions were performed in triplicate using 0.25 μl (5 U/μl) FastStart High Fidelity Taq DNA Polymerase (Roche, Basel, Switzerland), 2.5 μl (10×) FastStart High Fidelity reaction buffer without MgCl_2_, 2.3 μl (25 mM) MgCl_2_, 0.5 μl (10 mM) PCR nucleotide mix, 0.25 μl (20 mg/ml) bovine serum albumin, 0.5 μl of each (10 mM) primer and 10 ng of sample DNA. The thermal cycling protocol was 95 °C for 5 min, 30 cycles of 95 °C for 40 s, 58 °C for 45 s, 72 °C for 35 s and a final extension of 10 min at 72 °C. All amplicons were run in an agarose gel (1 % *w*/*v*) and bands containing exact sizes were excised from the gel and purified using the QIAquick Gel Extraction Kit (QIAGEN, Hilden, Germany). Purified amplicons from each triplicate reaction were pooled together in order to minimize PCR bias and then sequenced at Genewiz (South Plainfield, NJ, USA).

Sequencing raw data were demultiplexed and processed using the Quantitative Insights Into Microbial Ecology toolkit (QIIME) (Caporaso et al. 2010a). The bacterial 16S rRNA gene partial sequences were then quality-trimmed using the following parameters: quality score >25 and sequence length >300 and <900 bp. The quality reads were then binned into operational taxonomic units (OTUs) at 97 % sequence identity using UCLUST (Edgar 2010). A representative sequence for each OTU was aligned against the Greengenes coreset (DeSantis et al. 2006) using PyNAST (Caporaso et al. 2010b); then, the sequences were taxonomically classified using the Greengenes database via the RDP classifier (Wang et al. 2007). For all OTU-based analyses, the original OTU table was rarified to a depth of 8500 sequences per sample (the fewest in a single sample). Moreover, QIIME was also used to generate weighted UniFrac distance matrices.

### Metagenome sequencing and processing of unassembled sequences

The DNA samples from the microbial consortia (in triplicate) cultivated on the once-used plant biomass (WS1-M, SG-M and CS-M; *n* = 9) were subjected to Illumina MiSeq v2 sequencing (250 bp paired-end reads) at LGC Genomics (Berlin, Germany). Overlapping sequence pairs were matched, and non-overlapping reads retained as individual reads, after which dereplication was performed. Duplicate read based inferred sequencing error estimation and quality trimming (phred score <20) was done using default settings in MG-RAST v3.1.2 server (Meyer et al. 2008). Gene predictions were done using the FragGeneScan software and subsequently, the predicted proteins were annotated based on BLASTX searches against the RefSeq database using an *e* value cutoff of 1e-15, a minimum alignment length of 50 amino acids and a minimum identity of 50 % (Jiménez et al. 2015a). All metagenome sequences are publically accessible on the MG-RAST server (Metagenome IDs 4579476.3 to 4579481.3 and 4579485.3 to 4579487.3).

### Taxonomic affiliation of unassembled sequences and profiles of bacterial genes involved in polysaccharide deconstruction

For the interpretation of the overall microbial structure, the RefSeq database was accessed to identify protein-encoding sequences. The taxonomic read assignment was performed by the Lowest Common Ancestor (LCA) algorithm and the representative hit classification in MG-RAST. To evaluate the relative abundance (RA) of reads per bacterial genus, the read counts were normalized using the total numbers of quality reads matched in the RefSeq database per metagenome. Genera with ≥2 % of RA, in the datasets, were used to perform principal components analysis (PCA) in the R platform v2.15 (R development Core Team 2011). Carbohydrate-active enzymes were detected using, as a starting point, the unassembled reads (quality-filtered and trimmed) obtained by MG-RAST. Annotation was performed via Hidden Markov Models based on CAZy family domains (v3) (downloaded from dbCAN site) (http://csbl.bmb.uga.edu/dbCAN/) (Yin et al. 2012) using an *e* value cutoff of 1e-15. Bacterial glycosyl hydrolase (GH) families involved in polysaccharide deconstruction were selected according to Berlemont and Martiny (2015). To evaluate the RA of reads per selected GH family, the counts were normalized to hits, or unique matches, per million reads, thereby accounting for differences in metagenome sizes (Cardenas et al. 2015). Heat maps were constructed in the R platform v2.15 using the row *Z* score for each GH family. In addition, correlation (*r* ^2^) values of the taxonomic (genus level) and CAZy family profiles across all metagenomes were obtained using the STAMP package (Parks and Beiko 2010).

### Analysis of polysaccharide-degrading enzymes in the consortial metasecretomes

Extractions of the extracellular protein fractions (metasecretome) from each microbial consortium (WS1-M, SG-M and CS-M) were performed after 6 days of growth (transfer 13). The enrichment cultures were centrifuged (12,000*g*, 10 min) (Eppendorf minicentrifuge, Hamburg, Germany) and the supernatants passed through 0.22-μm syringe filters (Whatman FP30/0.22 - cellulose acetate membrane, Little Chalfont, UK). Quantification of the proteins was performed by the Bradford assay. In order to evaluate plant biomass-degrading endo-activities in the secreted fraction of each microbial consortium, we used a new generation of versatile chromogenic substrates (Kračun et al. 2015) (supplied by GlycoSpot IVS, Farum, Denmark). Briefly, nine chromogenic polysaccharide hydrogels (CPH) and three insoluble chromogenic biomass (ICB) substrates were evaluated (Table 1). The CPH substrates were used in a 96-well filter plate, where the solid CPH were activated by adding 200 μl of sterile water and incubating for 15 min. Then, the water was removed by centrifugation (2700*g*, 10 min), and the plate washed again with water to remove free dye. For the ICB substrates, 3 mg (50 μl of 3 g/50 ml in isopropanol) was transferred into each well, after which the wells was washed with water to remove the isopropanol and free dye. The reaction mixture consisted of 150 μl of 100 mM Na-phosphate buffer (pH 7.0) and 5 μl of each supernatant (adjusted to approximately 0.3 mg of total proteins/ml). Three biological replicates (flasks) of each microbial consortium were used. The plastic lid was put on top of the reaction plate and incubated for 24 h at 30 °C and 150 rpm. Then, the supernatant was transferred by centrifugation into the collection plate. The absorbances at 517 nm (red) and 630 nm (green) were determined using a plate reader. Positive controls for each substrate were also set up using commercial enzymes (supplied by Megazyme, Wicklow, Ireland) (final concentration of the positive control in each well: 0.1 U/ml) (Table 1). In addition, we used sterilized water as a negative control. Semi-quantitative data were obtained based on the absorbance values. Statistical comparisons between the absorbance values were performed using one-way ANOVA (Tukey’s test).

**Table 1:** Chromogenic substrates and positive controls used for detection of plant polysaccharide-degrading activities in the consortial metasecretomes

Polysaccharide (Kračun et al. 2015)	Colour	Enzyme-positive control (ID^b^)
CPH-2-hydroxyethylcellulose^a^	Green	Endo-β-1,4-D-glucanase^d^ (E-CELBA)
CPH-arabinan	Green	Endo-arabinase (E-EARAB)
CPH-arabinoxylan	Green	Endo-β-1,4-xylanase^e^ (E-XYNBCM)
CPH-galactomannan	Green	Endo-β-1,4-mannanase^e^ (E-BMACJ)
CPH-pullulan	Green	Pullulanase M1 (E-PULKP)
CPH-rhamnogalacturonan	Green	Pectate lyase^f^ (E-PECLY)
CPH-xylan	Green	Endo-β-1,4-xylanase^e^ (E-XYNBCM)
CPH-xyloglucan	Green	Xyloglucanase^c^ (E-XEGP)
CPH-β-glucan from barley	Green	Endo-β-1,3-glucanase (E-LAMSE)
ICB-baggasse	Red	Endo-β-1,4-xylanase^e^ (E-XYNBCM)
ICB-wheat straw	Red	Endo-β-1,4-xylanase^e^ (E-XYNBCM)
ICB-willow	Red	Endo-β-1,4-xylanase^e^ (E-XYNBCM)

^a^CPH-HE cellulose

^b^ID: Enzyme code (supplier Megazyme)

^c^From *Paenibacillus* sp.

^d^From *Bacillus* sp.

^e^From *Cellvibrio* sp.

^f^From *Aspergillus* sp.

## Results

### Community structures compared between the original and newly selected microbial consortia

Here, we used an innovative enrichment strategy, with two stages, based on partially degraded plant biomass as the carbon source. First, the original (soil-derived) microbial consortia were grown, in a sequential-batch approach, on untreated wheat straw, switchgrass and corn stover. Subsequently, the resulting consortia (WS1, SG and CS—transfer 9) were used as the source inocula for a second growth step. For this, we used washed and autoclaved plant biomass originating from the last transfer of the first enrichment. The second-phase consortia (newly selected: WS1-M, SG-M and CS-M—transfer 13) were thus obtained using three sequential transfers into fresh medium with sterile, once-used, plant biomass as the sole carbon source (Fig. 1a). PCR-DGGE based on the bacterial 16S rRNA gene diversity showed that the three second-phase enrichments were each distinct, revealing less than 50 % of band similarity with any of other two systems. Based on nMDS, we observed approximately 64 % dissimilarity between WS1 and WS1-M, 57 % dissimilarity between SG and SG-M and 77 % dissimilarity between CS and CS-M. In terms of “richness” (using the number of DGGE bands as the proxy), we did not observe large changes in the selected microbial consortia (transfer 13) compared with the original ones (transfer 9), as the richness values ranged from 8 to 10 abundant types across all consortia (Supplementary Fig. S1).

**Fig. 2:**
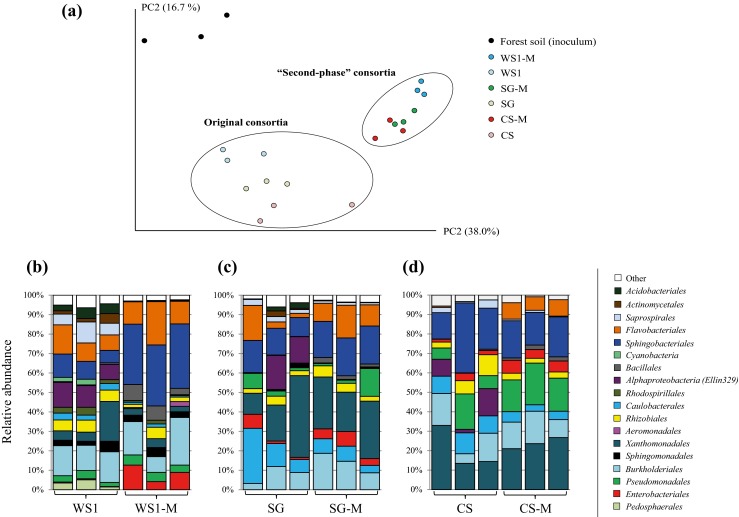
**a** Principal components analysis (PCA) of bacterial community structure, comparing the forest soil inoculum, the original (WS1, SG and CS) and the “second phase” microbial consortia (WS1-M, SG-M and CS-M). PCAs are shown for the weighted UniFrac community dissimilarity. **b**–**d** Bacterial community composition compared between the original and the second-phase microbial consortia. Classes with ≥2 % of relative abundance, in the datasets, were used to assemble the bar figures

We then performed bacterial 16S rRNA gene-based amplicon sequencing to the original and newly cultured consortia (Fig. 2). The data confirmed (based on weighted UniFrac distance matrices) that the bacterial community structures of the forest soil (inoculum), the original and the second-phase consortia were quite dissimilar (Fig. 2a). In terms of composition, we noted that organisms falling in the classes *Enterobacteriales*, *Pseudomonadales*, *Flavobacteriales*, *Bacillales* and *Burkholderiales* were enriched in all second-phase consortia compared with the original ones (Fig. 2b–d). In addition, *Sphingobacteriales* were most abundant in the WS1-M (31.5 ± 0.9 %) and SG-M (18.8 ± 0.4 %) compared with the WS1 (8.4 ± 2.3 %) and SG (12.7 ± 2.9 %) consortia, respectively. Moreover, *Xanthomonadales*, although slightly most abundant in SG-M (25.0 ± 3.7 %) and CS-M (23.1 ± 2.2 %) compared with the SG (22.3 ± 12.1 %) and CS (18.5 ± 8.2 %) consortia, remained at a rather similar relative abundances. The data also showed that the relative abundances of the classes *Actinomycetales*, *Acidobacteriales*, *Caulobacteriales*, *Saprospirales* and of the *Alphaproteobacteria* (Ellin329) group decreased by the second enrichment process in all three microbial consortia (Fig. 2b–d).

### Lignocellulose degradation rates

Substrate weight loss was evident in the three sequential transfers (11, 12 and 13) of the selected microbial consortia. Consortium CS-M showed a substrate consumption between 47 and 49 %, while WS1-M and SG-M revealed approximately 42–45 % plant biomass consumption (Supplementary Fig. S2). FTIR spectroscopy was used to evaluate the proportions of lignin, cellulose and hemicellulose in the untreated and once-used substrates. In addition, we analysed the remains of the plant biomass (twice used) after growth of the newly selected microbial consortia (Fig. 1b). Thus, degradation rates of lignin, cellulose and hemicellulose were obtained (Table 2).

**Table 2:** Degradation rates of lignin, hemicellulose and cellulose across the microbial consortia

Consortia	Degradation rates (%)				
	Lignin	Cellulose	Hemicellulose	Substrate	Reference
WS1	18.8 ± 1.5	51.9 ± 0.3	48.5 ± 0.9	Original	Brossi et al. (2015)
SG	39.3 ± 3.3	48.0 ± 1.6	54.6 ± 3.6	Original	
CS	31.0 ± 1.9	37.4 ± 0.1	62.8 ± 3.8	Original	
WS1-M	25.3 ± 1.8	31.7 ± 1.2	28.7 ± 2.9	Once used	This study
SG-M	58.6 ± 1.0*	20.5 ± 2.3	21.8 ± 0.8	Once used	
CS-M	24.7 ± 1.2	57.7 ± 1.7*	42.0 ± 2.4*	Once used	

*Significantly higher than (all) other corresponding values; ANOVA Tukey’s pairwise test *p* < 0.01

Lignocellulose was consumed by both the original and the newly selected consortia. Regarding those cultivated on wheat straw, the degradation rates of cellulose (51.9 ± 0.3 %) and hemicellulose (48.5 ± 0.9 %) were higher in the original consortium (WS1) compared to the selected one (WS1-M). In addition, the SG consortium also showed higher degradation rates of cellulose and hemicellulose (48.0 ± 1.6 and 54.6 ± 3.6 %, respectively) than SG-M. In the CS consortium, the degradation rate of cellulose was 37.4 ± 0.1 %, whereas the CS-M one showed a rate of 57.7 ± 1.7 %, suggesting an increased availability of cellulose on the once-used corn stover as compared to the untreated substrate. However, hemicellulose degradation rates were higher in CS (62.8 ± 3.8 %) compared with CS-M (42.0 ± 2.4 %) (Table 2).

In the WS1-M consortium, the degradation rates of lignin, cellulose and hemicellulose were similar, approximately ranging from 25 to 30 %. Of the three consortia, the SG-M one was the most effective in the degradation of lignin (58.6 ± 1.0 %) (*p* < 0.01). Notably, the degradation rates of cellulose and hemicellulose in CS-M were higher than those obtained with WS1-M and SG-M (*p* < 0.01) (Table 2).

### Metagenomics-based analysis of the microbial consortia cultivated on once-used plant biomass

**Fig. 3:**
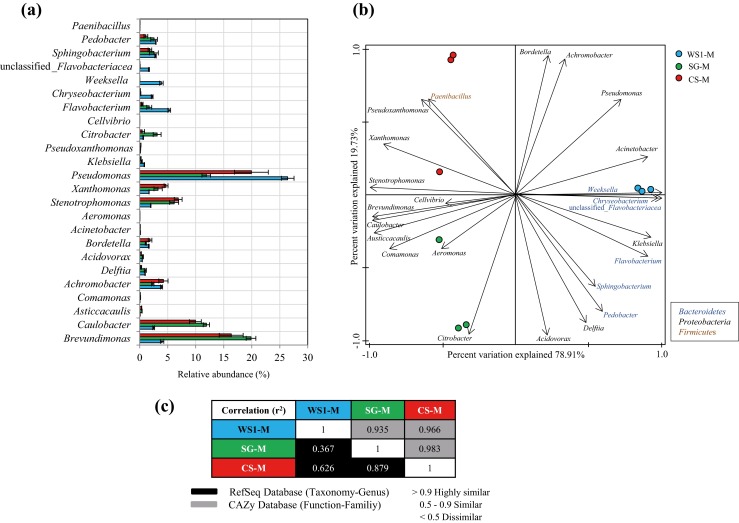
**a**, **b** Relative abundances (%) and principal components analysis of the most abundant genera (>2 %) across the WS1-M, SG-M and CS-M microbial consortia. **c** Correlation (*r* ^2^) between the taxonomic profile and the carbohydrate-active enzyme profile across the WS1-M, SG-M and CS-M microbial consortia

Approximately 4.9 Gb of metagenomic information was obtained from the three selected microbial consortia (1.5, 1.6 and 1.8 Gb for WS1-M, SG-M and CS-M, respectively). Based on the LCA algorithm, 48.6 ± 1.69 (SG-M) to 50.3 ± 0.94 % (WS1-M and CS-M) of the total sequences were affiliated with sequences from the domains *Eukarya*, *Bacteria* or *Archaea*. Of these, >99 % was affiliated with genes from bacterial genomes. We used all identifiable protein-encoding sequences to infer their origin and so host abundance. On the basis of the total coding regions, the most abundant genus in WS1-M was *Pseudomonas* (26.41 ± 1.13 %), followed by *Flavobacterium* (5.27 ± 0.23 %), *Brevundimonas*, *Achromobacter* and *Weeksella* (around 4 %). Regarding the CS-M and SG-M consortia, *Pseudomonas* (19.93 ± 3.01 and 11.88 ± 0.76 %), *Brevundimonas* (16.33 ± 2.13 and 19.86 ± 0.87 %) and *Caulobacter* (9.94 ± 1.04 and 11.90 ± 0.55 %) stood out as abundant coding genera (Fig. 3a). The PCA performed on these data showed three major groups. The first group encompassed all WS1-M, the second all CS-M and the third all SG-M consortia (Fig. 3b). The CS-M and SG-M consortia were placed closely together in the biplot, suggesting rather similar structures between them. *Bacteroidetes* (*Chryseobacterium*, *Weeksella, Flavobacterium*, *Sphingobacterium* and *Pedobacter*); *Klebsiella*; *Acinetobacter*; *Pseudomonas*; *Bordetella*; *Achromobacter*; *Delftia* and *Acidovorax* were preferentially selected in WS1-M. In contrast, in SG-M and CS-M, *Proteobacteria*, i.e. *Citrobacter*, *Aeromonas*, *Comamonas*, *Austiccacaulis*, *Caulobacter*, *Brevundimonas* and *Cellvibrio* were dominant (in SG-M); this, next to *Stenotrophomonas*, *Xanthomonas* and *Pseudoxanthomonas* (in CS-M). A *Firmicutes* genus, *Paenibacillus*, was preferentially selected on CS-M (Fig. 3b). These coding-gene-based data corroborate the bacterial 16S rRNA gene amplicon sequencing data (Fig. 2), and add a new dimension to these, i.e. a more detailed vision of the total (dominant) genomes present in the consortia.

### Functional redundancy and metabolic potential to deconstruct plant polysaccharides

Correlation analysis, using taxonomic (RefSeq database) and functional annotation (CAZy database) of the protein-encoding sequences, showed that the WS1-M consortium was taxonomically dissimilar (*r* ^2^ < 0.8) from the SG-M and CS-M ones (*r* ^2^ = 0.879; similarity between them). However, the three selected microbial consortia showed a highly similar functional profile in terms of the plethora of carbohydrate-active enzyme families that were present (*r* ^2^ > 0.93) (Fig. 3c). However, the high percentage (~50 %) of sequences with no or negligible homology to any database sequence could mask the differences between the functional profiles across the microbial consortia.

**Fig. 4:**
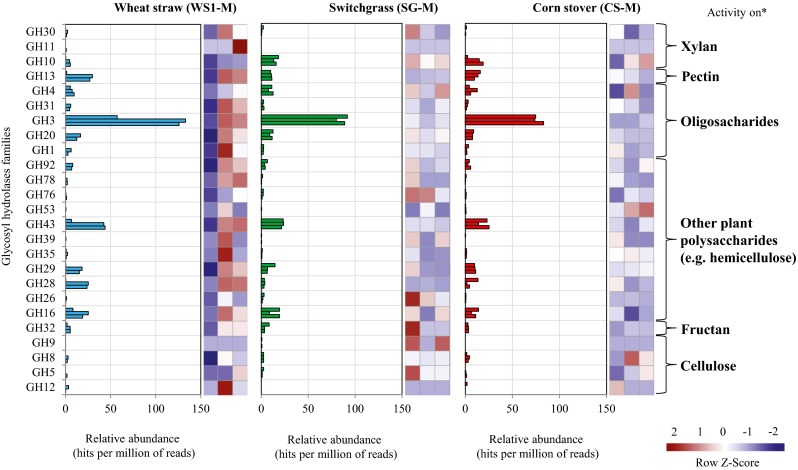
Relative abundance (hits per million of reads) of the GH families involved in plant-polysaccharide deconstruction across the WS1-M, SG-M and CS-M microbial consortia. Data are represented by triplicate flasks. *Asterisk* represent mode of action of each family according to Berlemont and Martiny (2015). Heat maps were constructed using the row *Z* score and comparing the three microbial consortia

Across the metagenomes, genes encoding proteins of CAZy families GH3, GH43, GH13, GH10, GH29, GH28, GH16, GH4 and GH92 were most prevalent (approx. >10 hits per million of reads). In contrast, genes for enzymes involved in cellulose degradation (e.g. endoglucanases—GH5) were found in low abundance (approx. <1 hit per million reads). The GH3 family, which contains proteins that can act on (hemi)cellulose structures, was found to be highly abundant (Fig. 4). Based on the recently discovered relevance of redox enzymes for the degradation of plant biomass (specifically cellulose and lignin), we analysed the profile of auxiliary activities (AA) using the CAZy database. The results showed that the most abundant AA families in the WS1-M, SG-M and CS-M consortia were AA6 (1,4-benzoquinone reductases) and AA10 (LPMOs), followed by the low-abundance families AA2 (lignin peroxidases), AA7 (gluco oligosaccharide oxidases) and AA4 (vanillyl-alcohol oxidases) (Supplementary Fig. S3).

### Degradation of plant polysaccharides by secreted enzymes and oligosaccharide production

**Fig. 5:**
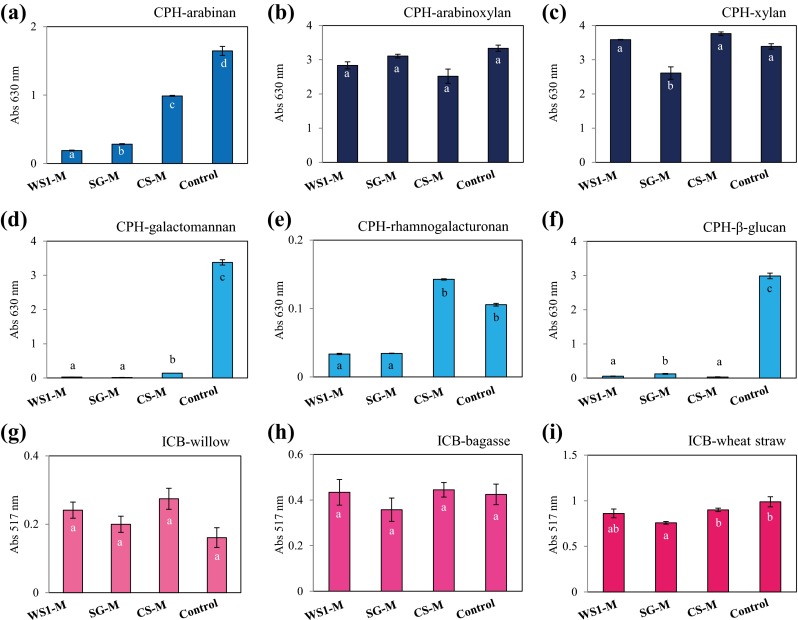
Absorbance values obtained from the product plates used to evaluate the secreted enzymatic activity of WS1-M, SG-M, CS-M and the positive controls (Table 1) on nine chromogenic polysaccharide hydrogels (CPH) and three insoluble chromogenic biomass (ICB) substrates. *Different lowercase letters* refer to differences among absorbance values across the microbial consortia with each chromogenic substrate (ANOVA, *p* < 0.01)

The potential to deconstruct plant polysaccharides was evaluated in the three selected microbial consortia using recently developed chromogenic substrates that mimic complex polysaccharides and plant biomass. The activity of the secreted endo-enzymes after growth on each plant biomass (transfer 13) was evaluated using nine CPH and three ICB substrates. CPH substrates are made from defined polysaccharides, whereas ICB substrates are coloured versions of native biomass containing complex mixtures of polysaccharide. Enzymatic activity of the metasecretomes was detected on all tested substrates, except CPH-cellulose, CPH-pullulan and CPH-xyloglucan (Supplementary Fig. S4). The highest activities were observed on CPH-xylan and CPH-arabinoxylan. Interestingly, high enzymatic activity was also found on ICB-wheat straw, ICB-bagasse and ICB-willow (Fig. 5). The CS-M consortial metasecretome showed highest activity on CPH-arabinan, CPH-galactomannan and CPH-rhamnogalacturonan, in contrast to significantly lower activities of WS1-M and SG-M (*p* < 0.01). The SG-M consortium showed high activity on CPH-β-glucan, but low activity on CPH-xylan compared with CS-M and WS1-M (*p* < 0.01). The enzymatic activities on the ICB substrates were similar in the three consortia, suggesting that they could have the same potential to degrade plant biomass. Compared with the positive controls, the consortial metasecretomes showed low activity with CPH-arabinan, CPH-galactomannan and CPH-β-glucan (*p* < 0.01) (Fig. 5).

## Discussion

Recent work has successfully enriched microbial consortia using plant waste as sole carbon source (Gladden et al. 2011a). Such microbial consortia were shown to have ample capacities to degrade plant biomass (D’haeseleer et al. 2013), being promising for lignocellulose saccharification (Park et al. 2012). Here, we used an innovative enrichment strategy, with two stages, based on partially degraded plant biomass (once-used) as sole carbon source. A similar approach was recently used in an anaerobic enrichment from lake sediment using switchgrass (Korenblum et al. 2016). Such a methodology is thought to enhance the prevalence of microbes acting on the most recalcitrant part of the lignocellulose (e.g. complex hemicellulose structures, crystalline cellulose and lignin). Additionally, this procedure may maintain plant biomass complexity, which decreases upon chemical and/or enzymatic pretreatments due to generation of more defined substrates (Lazuka et al. 2015). Clearly, a better picture of the consortial behaviour will be obtained by the evaluation of the time points along the microbial growth and incubation. However, it is known that the expression and secretion of the enzymes involved in the lignocellulose degradation are often more frequent at the final stages of growth (start of the stationary phase). Based on this premise, we decide to compare across three microbial consortia at one end-point of incubation (6 days), that is, between the exponential and stationary phase of growth.

Based on the results, we postulated that substrate type is the main driver of the structure of microbial consortia developing in enrichments. Recently, Cortes-Tolalpa et al. (2016) reported that inoculum source is also a key factor that strongly influences the composition of plant biomass-degrading microbial consortia. However, stochastic factors (“first come, first bite”) might also have affected the selection process and so driven the microbial diversity in the consortia. Then, the growth on the partially degraded plant biomass clearly changed the structure of the original consortia (Fig. 2a; Supplementary Fig. S1), suggesting that substrate structure and composition indeed drove the communities. In this respect, different proportions of lignin, cellulose and hemicellulose were observed after the first growth step as compared to the untreated plant biomass (Fig. 1b). Regarding lignocellulose utilization, the degradation rates of hemicellulose were higher in the original microbial consortia compared with the selected ones, suggesting a higher availability of hemicellulose in the original substrates. Thus, this polymer could support, to a large degree, the growth of the consortia.

Here, we used 16S rRNA-based PCR-DGGE coupled to 16S rRNA gene amplicon sequencing in order to determine the bacterial community structures along the enrichment experiment. In this respect, distinct structures were observed between the microbial source and the first and second enrichment steps, suggesting that, indeed, the consortia were strongly driven by the nature of the substrate, i.e. fresh versus once-used (Fig. 2a). Although bacterial 16S rRNA gene (and fungal ITS1) surveys constitute powerful techniques to evaluate the diversity of microbial consortia (Jiménez et al. 2014b), the here used “gene-centric” metagenomics approach may be regarded as superior, since it allows for the simultaneous characterization of microbial community structure and its metabolic potential. The 16S rRNA gene and the total metagenomic data are complementary approaches. However, it is not possible to perform a direct comparison between them due to differences in numbers of 16S rRNA gene copies, the database used and the genome sizes between the consortium members. The metagenomics-based analyses were performed using unassembled sequences, as this is presumed to cause minimal disturbance with respect to the representation of sequences of the abundant genera in the dataset (Teeling and Glöckner 2012). Moreover, on the basis of previously reported ITS1 versus bacterial 16S rRNA gene copy numbers (Brossi et al. 2015), next to the annotation of our metagenomic sequences, we postulate that the microbial consortia were dominated by bacteria.

A comparison of the relative abundance values of the most abundant genera (>2 %) in our selected microbial consortia with the ones reported from forest soil metagenomics data (similar inoculum as used in this study; Jiménez et al. 2015a) showed a fold increase of approximately 200 and 165 for *Brevundimonas* spp. in SG-M and CS-M, respectively. In contrast, *Weeksella* was the most enriched genus in WS1-M (~350-fold increase) (Supplementary Fig. S5). These organisms were undetectable by culture-based approaches applied to the original consortia (Brossi et al. 2015), suggesting their preferential growth on the once-used plant biomass.

Based on the assumption that mainly microbes active in plant biomass degradation were enriched, it is reasonable to propose that such abundant consortium members contain enzymatic machineries that allow the deconstruction of lignocellulosic structures. The SG-M consortium that contained high abundances of *Brevundimonas*, *Caulobacter*, *Pseudomonas*, *Citrobacter* and *Aeromonas*, showed a high lignin degradation rate (~59 %). *Caulobacter-*like organisms were undetectable, by culture-based approaches, in the SG consortium (Brossi et al. 2015), which is consistent with a presumed selection of these microbes by the second growth step. However, the 16S rRNA gene amplicon-sequencing data showed a slight decrease of the abundance of *Caulobacteriales*-like organisms from the SG to SG-M consortia (Fig. 2c). Otherwise, DeAngelis et al. (2011a) reported enrichments of *Caulobacter* and *Brevundimonas* types (catalase producers) in lignin-amended soils compared with unamended ones. Moreover, it has been shown that *Pseudomonas* and *Aeromonas* have high capacities to transform lignin (Prabhakaran et al. 2015; Wu et al. 2010). For instance, Wang et al. (2013) reported a bacterial consortium that could break down 60.9 % of lignin in reeds at 30 °C under conditions of static culture within 15 days. This consortium was dominated by *Pseudomonas* species. In addition, Abhishek et al. (2015) showed that *Citrobacter freundii* can co-metabolize model and kraft lignin. These studies reflect the relevance of such taxa in lignin bioconversion by the SG-M consortium. Notably, *Pseudomonas* was the most abundant taxon in the WS1-M and CS-M consortia. However, the lignin degradation rates were significantly lower than those in the SG-M consortium (*p* < 0.01), suggesting that *Brevundimonas* and *Caulobacter* species in SG-M may be the more relevant lignin degraders. Considering the latter, it is still unclear whether the lignin was completely metabolized or is present as modified acid-precipitable polymeric lignin (a water-soluble catabolite) in the culture supernatant, as has been observed for a compost-derived microbial consortium cultivated on pretreated switchgrass (Eichorst et al. 2014). One possible reason for the high degradation of lignin in SG-M might relate to a lower lignin recalcitrance in switchgrass, as compared to wheat straw and corn stover. Alternatively, the SG-M consortium might have developed a higher synergism between the degraders.

In terms of cellulose and hemicellulose degradation, the CS-M consortium showed significantly higher degradation rates than the SG-M and WS1-M consortia (*p* < 0.01). This CS-M consortium was mostly composed of *Pseudomonas*, *Brevundimona*s and *Caulobacter* types, but members of *Stenotrophomonas*, *Xanthomonas*, *Pseudoxanthomonas*, *Achromobacter* and *Paenibacillus* were also preferably selected (Fig. 3b). Previous genome sequence analyses revealed that *Caulobacter crescentus* has the potential to degrade plant polysaccharides through the production of exo-enzymes, including cellulases, xylosidases and polysaccharide deacetylases (Nierman et al. 2001). Song et al. (2013) have shown degradation of cellulose by the mesophilic *Caulobacter* sp. FMC1 under aerobic and anaerobic conditions. Moreover, Eichorst and Kuske (2012) found that members of the *Caulobacteriales* and *Xanthomonadales* became prevalent in soil microcosms amended with [^13^C] cellulose. Besides, Talia et al. (2012) reported the presence of *Brevundimonas*, *Caulobacter*, *Pseudomonas*, *Xanthomonas*, *Stenotrophomonas*, *Achromobacter* and *Paenibacillus* species in carboxymethylcellulose (CMC) and filter paper enrichment cultures from soil. Additionally, several strains of *Pseudomonas*, *Stenotrophomonas* and *Paenibacillus* retrieved from the CS consortium showed CMC-ase activity (Brossi et al. 2015). These studies reinforce our results, suggesting that the CS-M microbial consortium contains key members that were highly relevant in the degradation of (hemi)cellulose.

In this study, the WS1-M consortium was dominated by *Pseudomonas* species that could be related with lignin bioconversion. As we also observed a strong selection of *Bacteroidetes* (e.g. *Flavobacteriales* and *Sphingobacteriales*) (Fig. 2b), similar to previous results (Jiménez et al. 2014b), these data suggest that polysaccharides present in wheat straw selected for *Bacteroidetes* instead of *Proteobacteria*. *Bacteroidetes* like *Sphingobacterium* species can secrete enzymes such as endo-β-1,4-xylanases, α-L-arabinofuranosidases, β-glucosidases, α-glucuronidases and α-L-fucosidases when grown in the presence of wheat straw (Jiménez et al. 2015b). Interestingly, organisms belonging to the *Enterobacteriales* (e.g. *Klebsiella*, *Kluyvera* and *Enterobacter* species) were most abundant in the WS1-M consortium as compared with WS1. The high abundance of *Enterobacteriales* in WS1-M was in line with the high frequency of strains belonging to this class retrieved from WS1 (Brossi et al. 2015). This suggested that, in this scenario, key organisms of the *Enterobacteriales* are strongly involved in the deconstruction of complex and recalcitrant plant polysaccharides. Degradation of lignin by *Enterobacter* and *Klebsiella* species has indeed been reported in recent papers (DeAngelis et al. 2011b; Woo et al. 2014).

Regarding the carbohydrate-active enzyme profiles, CAZy families GH10 (endoxylanases), GH3 and GH43 contain enzymes mainly involved in xylan, arabinan or arabinoxylan degradation, whereas families GH13 and GH28 are often active on pectin and rhamnogalacturonan, respectively. In addition, families GH3 and GH4 have broad substrate specificities and proteins of these families have β-D-glucosidase (GH3 and GH4), *N*-acetyl-β-D-glucosaminidase (GH3), α-glucosidase, α-galactosidase and α-glucuronidase (GH4) activities. The GH3 family was found to be highly abundant (Fig. 4). Similar results were reported in a rice straw-degrading microbial consortium (Wongwilaiwalin et al. 2013). Moreover, family GH16 enzymes cleave β-1,4 or β-1,3 glycosidic bonds in various glucans and galactans. Finally, families GH29 (α-L-fucosidases) and GH92 (α-mannosidases) contain exo-acting enzymes that can release fucose and mannose, respectively, from hemicellulose structures. Based on these considerations, we suggest that the three selected microbial consortia contain a wide genomic capacity to deconstruct different classes of plant polysaccharides, including hemicellulosic polymers.

Although relative gene abundances do not report on actual enzymatic activities, we found relations between the abundance of particular metabolic potential (in terms of GH relative abundances) and the defined extracellular enzymatic activities. For example, high frequencies of genes encoding proteins of CAZy families GH10, GH3, GH43, GH28 and GH16 were found in the WS1-M, SG-M and CS-M metagenomes. Proteins of these families could be related to the enzymatic activities detected on CPH-xylan, CPH-arabinan, CPH-arabinoxylan, CPH-rhamnogalacturonan, CPH-galactomannan and CPH-β-glucan. Moreover, the low abundance of enzymes involved in cellulose (e.g. CBHs and endoglucanases) and lignin degradation (e.g. AA2) is not a signal that the underlying genes cannot be expressed. However, we did not find endo-activity on CPH-HE-cellulose, suggesting that the xylo-oligosaccharides released from the hemicellulose structures could strongly inhibit the activity of endoglucanases (Kont et al. 2013). Alternatively, endo-cellulases might be more active at lower pH, where we have tested only at pH 7.0. Notably, Jiménez et al. (2014b) also reported a low activity of CBHs, compared with β-xylosidases and β-galactosidases, in the metasecretome of microbial consortia cultivated on wheat straw. Thus, the high activity of endo/exoglucanases, in plant biomass-degrading microbial consortia, may not be common. For instance, Gladden et al. (2011b) found low activities of CBHs and β-glucosidases in a microbial consortium bred on acid-pretreated switchgrass. Also, D’haeseleer et al. (2013) reported the absence of CBHs in the metasecretome of a thermophilic bacterial consortium adapted to deconstruct switchgrass. Indeed, the majority of secreted GHs were associated with the deconstruction of hemicellulose (e.g. GH3, GH10 and GH51) or α-glucan polysaccharides (GH13 and GH31). Moreover, genes for enzymes of families GH5 and GH9 (endoglucanases) were highly abundant in a mesophilic cellulose-converting consortium (Wang et al. 2015). Based on these studies, we posit that the low abundance of genes for CBHs and endoglucanases in our metagenomes, next to the low activities in the metasecretomes, are in some way related with the differential response to the composition of the substrate (Gladden et al. 2011b).

There is increasing interest in auxiliary enzymes acting on cellulose by a non-hydrolytic mechanism of depolymerization. Among these enzymes, LPMOs (CAZy family AA10) represent the most promising class due to their capability of enhancing the efficiency of lignocellulose degradation by acting on polysaccharides that are recalcitrant to cellulases within highly crystalline cellulose (Dimarogona et al. 2013; Beeson et al. 2015). With the recent discovery of AA10 enzymes, a new model for enzymatic cellulose depolymerization has been proposed. Thus these enzymes, which oxidatively cleave endoglycosidic bonds in crystalline cellulose, may create new chain ends that can be attacked by CBHs and this synergistic effect probably improves the overall hydrolysis yield (Horn et al. 2012). The presence of this gene type in all three consortia provides evidence of the capacity to degrade cellulose or increase the deconstruction of other plant polysaccharides by this new oxidative mechanism. Regarding the AA6 family, these are intracellular enzymes involved in the biodegradation of aromatic compounds. Benzoquinone reductases are involved in a quinone redox cycle that generates extracellular Fenton reagents. In addition, these enzymes are involved in lignin degradation by fungi (Levasseur et al. 2013; Dashtban et al. 2010). However, we still do not know the actual role of these proteins in a lignocellulolytic bacterium dominated consortium.

Finally, the production of oligosaccharides from plant biomass was detected using the ICB substrates. These findings suggest that the WS1-M, SG-M and CS-M microbial consortia have a high capacity to deconstruct plant biomass and convert complex polysaccharides into oligo and/or monosaccharides useful for downstream applications. The enzymatic activities detected on CPH and ICB substrates (Fig. 5) allowed to catalogue the three consortia as microbial enzyme “factories” that constitute excellent sources of efficient enzyme cocktails for the saccharification of plant biomass. Future experiments that combine the metasecretomes with available commercial cellulases can assist in raising the efficiency of plant biomass degradation for second-generation biofuel production. In addition, metatranscriptomics, metaproteomics and two-dimensional nuclear magnetic resonance spectroscopy (2D-NMR) analyses would help to better understand the lignocellulose degradation process (in particular the lignin bioconversion).

## Electronic supplementary material

ESM 1 (PDF 311 kb)
