# Supplementary material for: Characterization of three plant biomass-degrading microbial consortia by metagenomics- and metasecretomics-based approaches
Source: Appl Microbiol Biotechnol. 2016 Jul 14;100(24):10463–77. doi: 10.1007/s00253-016-7713-3 (PMC5119850; doi:10.1007/s00253-016-7713-3)
Supplement: Supplementary file 1 — (PDF 311 kb) [file 253_2016_7713_MOESM1_ESM.pdf]

Applied Microbiology and Biotechnology

Supplementary Material

Characterization of three plant biomass-degrading microbial consortia by metagenomics- and metasecretomics-based approaches

Diego Javier Jiménez [a *], Maria Julia de Lima Brossi [a], Julia Schückel [b], Stjepan Krešimir Kračun [b], William George Tycho Willats [b] and Jan Dirk van Elsas [a]

Affiliations
[a] Department of Microbial Ecology, Groningen Institute for Evolutionary Life Sciences, University of Groningen. Nijenborgh 7, 9747AG. Groningen, The Netherlands.
[b] Department of Plant and Environmental Sciences, University of Copenhagen. Thorvaldsensvej 40, Frederiksberg C 1871. Copenhagen, Denmark.

*Address correspondence to Diego Javier Jiménez. Email: d.j.jimenez.avella@rug.nl; Phone: +31(0)503632191; Fax: +31(0)503632348.

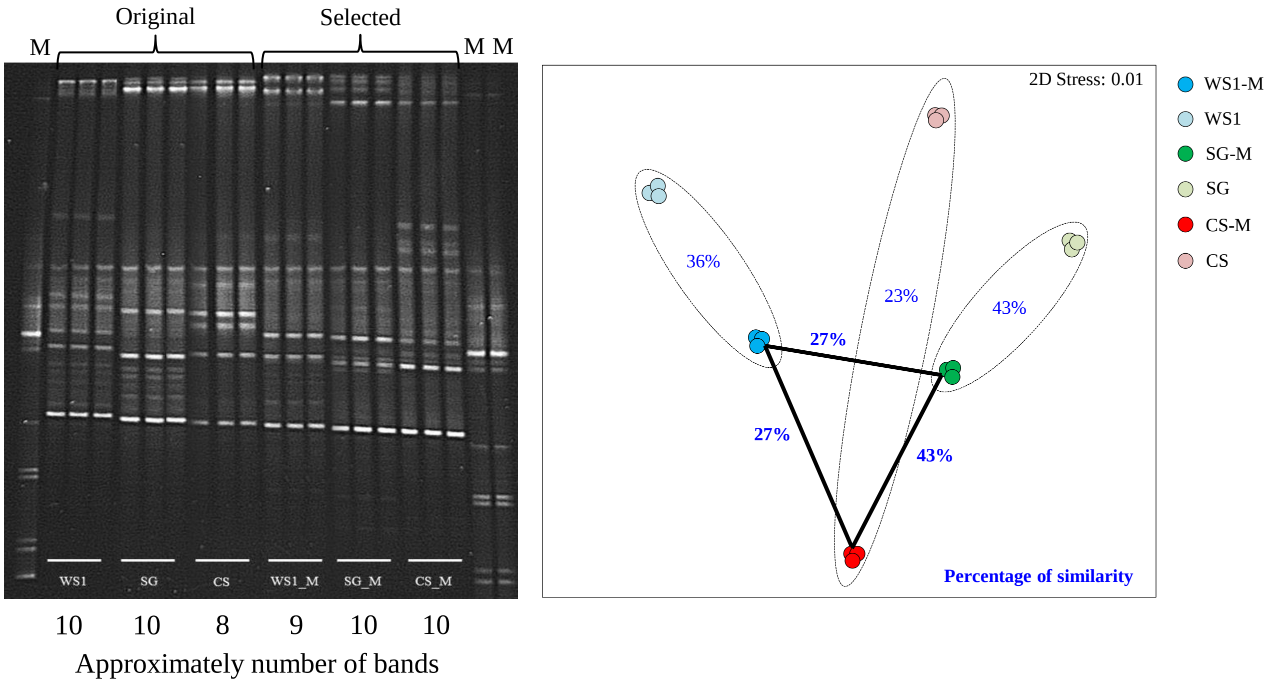

Supplementary Fig S1. 16S rRNA gene bacterial PCR-DGGE cluster analyses comparing soil-derived microbial consortia (original: WS1, SG, CS) with microbial consortia bred on once-used plant biomass (selected: WS1-M, SG-M and CS-M). M means DGGE markers.

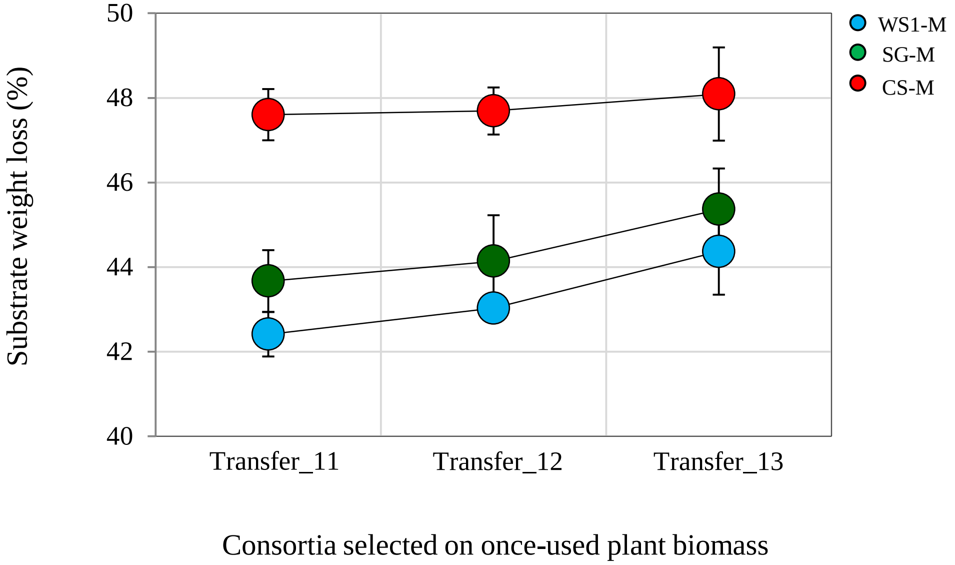

Supplementary Fig S2. Weight loss (%) of different substrates along the transfers 11, 12 and 13

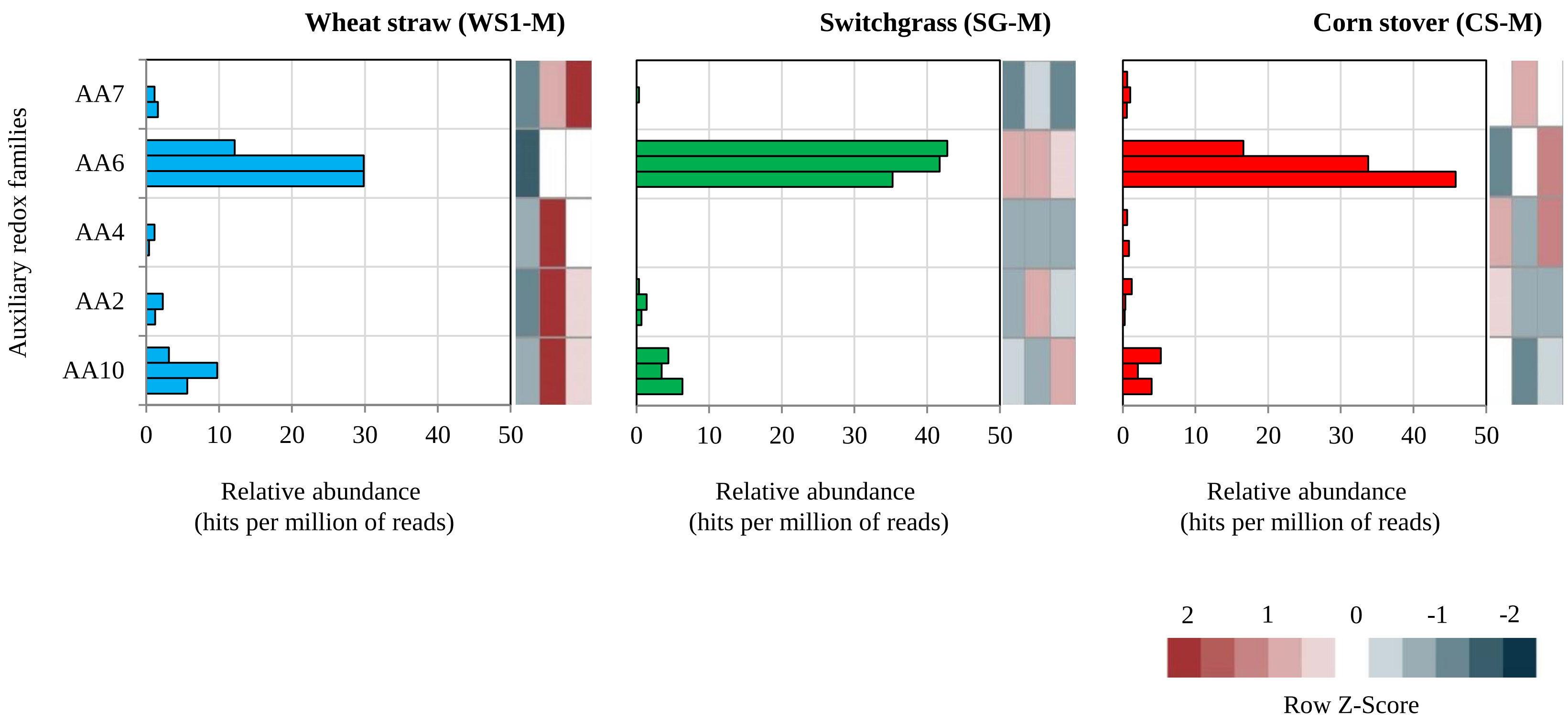

Supplementary Fig S3. Relative abundance (hits per million of reads) of the AA families across the WS1-M, SG-M and CS-M microbial consortia. Data is showed by triplicate flasks. Heat maps were constructed using the row Z-score and comparing the three microbial consortia.

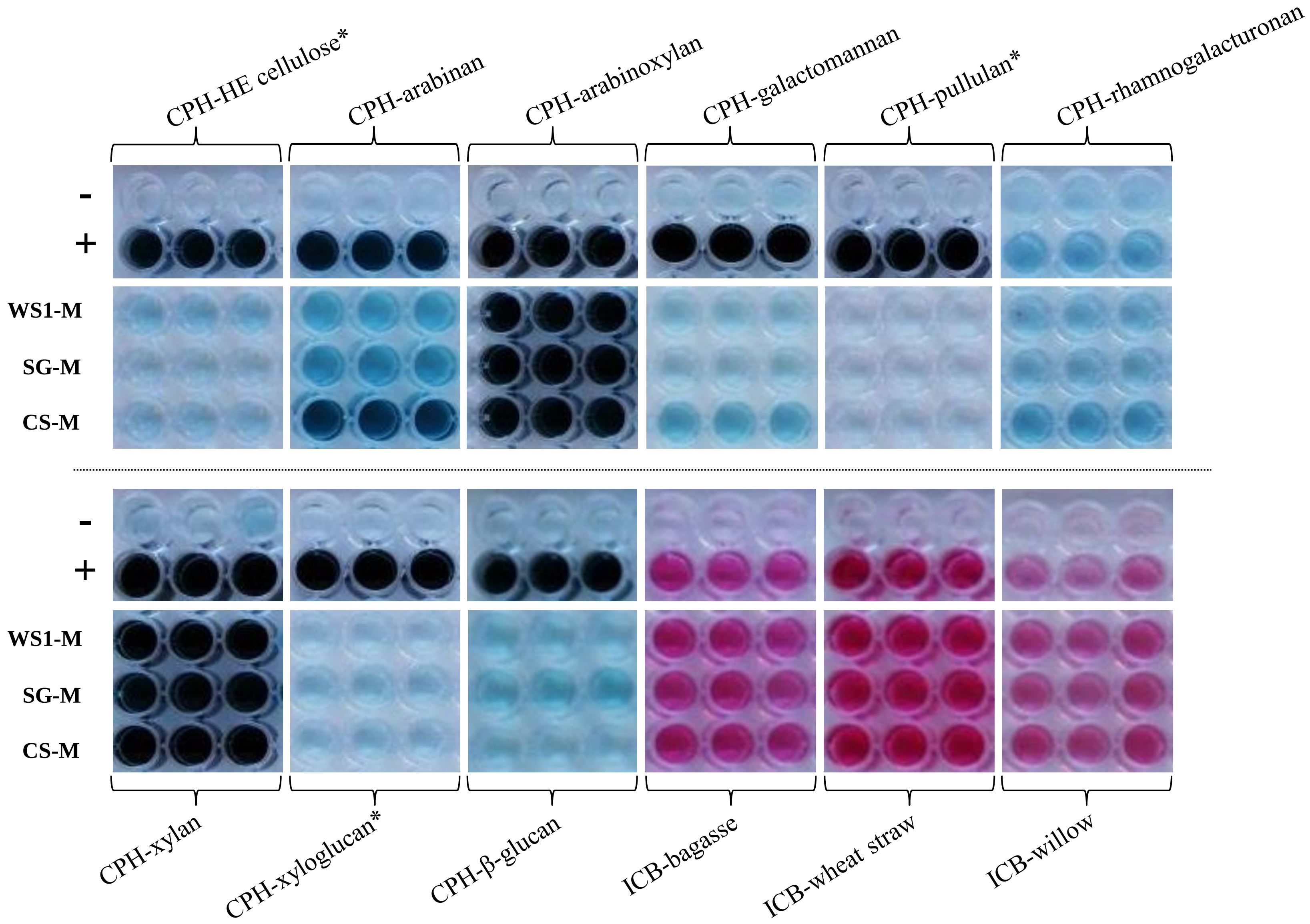

Supplementary Fig S4. Representation of the product plates used to evaluate the secreted enzymatic activity of WS1-M, SG-M and CS-M on nine chromogenic polysaccharides hydrogels (CPH) and three insoluble chromogenic biomass (ICB) substrates. +: positive controls, -: negative control. Asterisks represent substrates in which enzymatic activities were not observed.

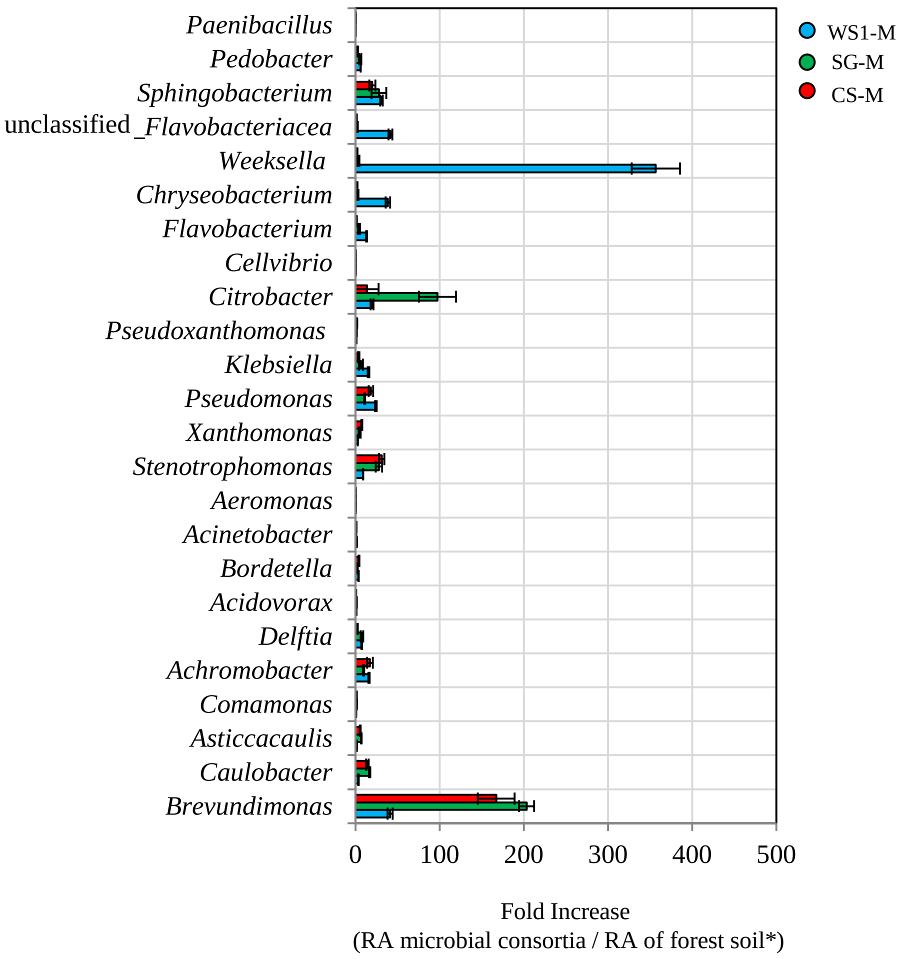

Supplementary Fig S5. Fold increase values (relative abundance microbial consortia / relative abundance of forest soil). Asterisk: The relative abundance of each genus was obtained from a forest soil metagenome (Jiménez et al. 2015a) using the same parameters of this study.
